# Competition and cooperation between active intra-network and passive extra-network transport processes

**DOI:** 10.1038/srep05269

**Published:** 2014-06-12

**Authors:** Dan Maruyama, Michal Zochowski

**Affiliations:** 1 Department of Physics, University of Michigan, Ann Arbor, MI 48109; 2 Biophysics Program, University of Michigan, Ann Arbor, MI 48109

## Abstract

Many networks are embedded in physical space and often interact with it. This interaction can be exemplified through constraints exerted on network topology, or through interactions of processes defined on a network with those that are linked to the space that the network is embedded within, leading to complex dynamics. Here we discuss an example of such an interaction in which a signaling agent is actively transported through the network edges and, at the same time, spreads passively through space due to diffusion. We show that these two processes cooperate or compete depending on the network topology leading to complex dynamics.

Biological, social, and physical networks can be natural or man-made. Understanding the relationship between their structure and their dynamics is of great scientific interest^1^,^2^,^3^. Some of these networks can be studied in isolation because they interact very weakly with the space or the environment that they are embedded within. A citation network is one example of such a network - the physical location of the authors does not affect the citation of their article. Other networks, however, are strongly affected by the physical space they sit in. These are usually referred to as spatial or geographical^4^ networks. They are subject to constraints stemming from the interaction with the space. This interaction can take different forms, the most common one being constraints, dependent on the metric of space, imposed on the network topology. This could be a cost function which creates longer connections or a distance dependent probability of connecting to other network elements^5^,^6^,^7^. Another type of spatial interaction can be defined when the network topology is independent of spatial (environmental) constraints, but the dynamics within the network involve other processes that are linked to or defined within the embedding space. In these instances transport mechanisms on the network interact with those defined on the environment. Other examples come to mind: neuronal or astrocytic networks that are coupled through neurites/gap junctions/synapses, but also secrete chemicals into the extracellular space, or epidemiological networks where the disease spreads through personal contact and through diffusion of pathogens through the air^8^.

In this paper we study an example of such an interaction. We define a chemical agent which spreads actively through the network but is also secreted into the “environment” where it undergoes passive spread governed by diffusion. Thus the transport is composed of an active component which allows for (nearly) instantaneous spread of the agent through the network, with the macroscopic pattern of its spread being dependent upon the network connectivity, and the passive spatial spread outside the network, through “environmental” diffusion. The interaction of these two transport processes, as we will show below, may be highly nonlinear and result in complex spatio-temporal dynamics of the network nodes.

## Results

**Figure 1:**
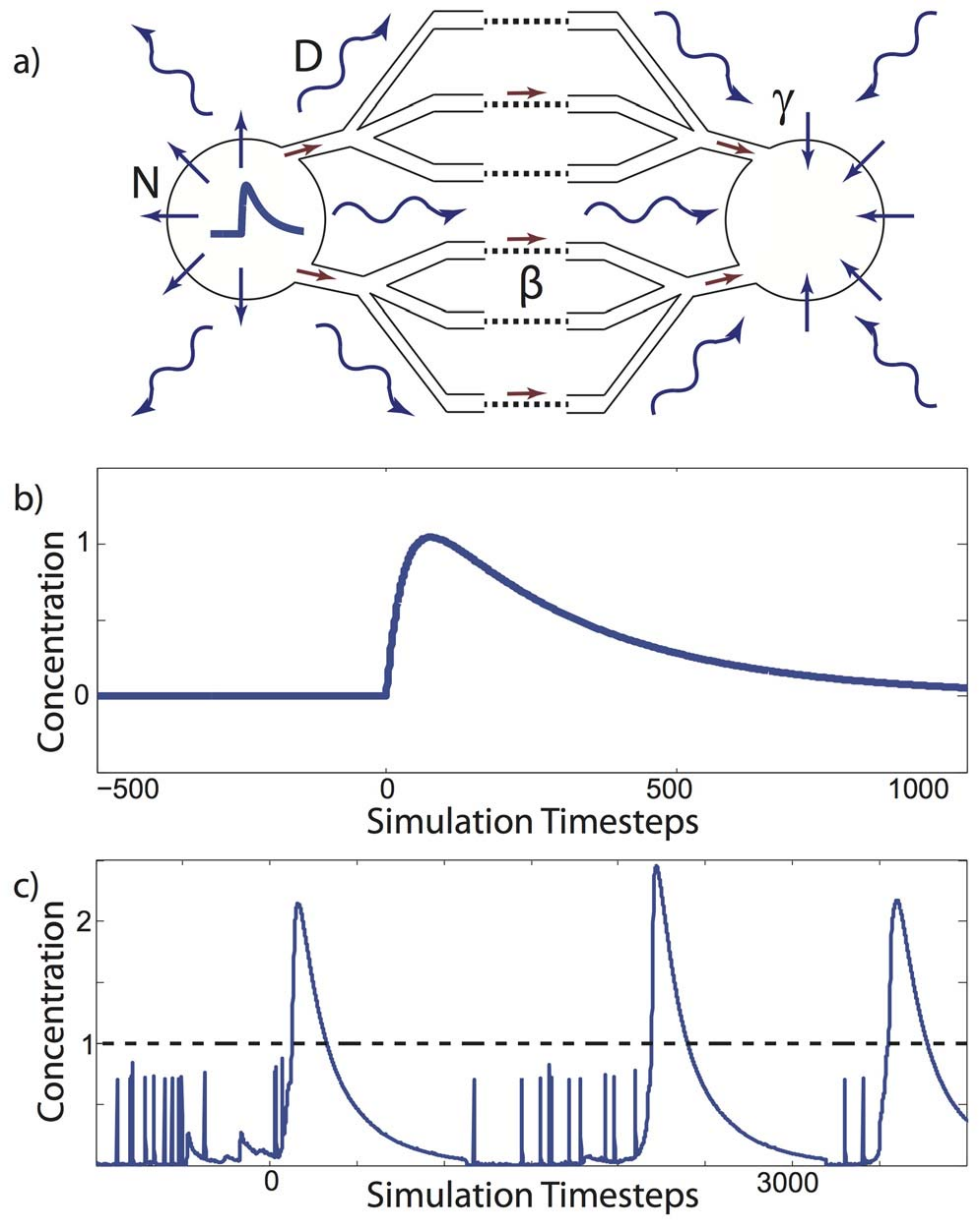
Description of the model. (a) Cartoon highlighting bimodal interactions from the active element (left) to the charging element (right). The processes underlying this interaction are 1) the active pathway through the network, defined by connectivity parameters (connectivity radius *R*, rewiring parameter *p*) and diffusive network coupling constant *β*, and, 2) the passive environmental diffusion, where *N* amount of the agent is emitted into the embedded space, diffuses subsequently with speed D* over to the charging element, which absorbs it with proportionality constant *γ*. (b) The time dependence of the release of the agent from internal nodal stores occurring after the node reaches the concentration threshold. (c) An example of the time evolution of agent levels within a single node; the small short spikes represent instantaneous changes in agent concentration due to noise, after the threshold (dashed line) is reached the node releases large amounts of agent into the network and its immediate environment.

We define a network composed of simple excitable integrate-and-fire elements (see Methods and Fig. 1 for a detailed description). When the level (or concentration) of an agent in the element exceeds a threshold, the element rapidly releases the agent from its internal stores (Fig. 1b) into the network and the surrounding physical space (Fig. 1a). The transport on the network is an active process - the signal spreads instantaneously to other nodes connected with the activated node through standard diffusive coupling. At the same time, the signal spreads passively via a spatial diffusion process, the nature of which is determined by the spatial extent and properties of the physical space, Fig. 1a. For simplicity, we consider that both these mechanisms only have positive signs - i.e they both spread excitation. This system loosely corresponds for example to astrocytic networks in the brain. Astrocytes, in simple terms, release calcium stored in the endoplasmic reticulum into the cytosol, which increases free calcium levels in the cell^9^. This calcium spreads through gap junctions to other astrocytes, but at the same time activates complex chemical cascades responsible for the release of glio-transmitters into the extracellular space. Glio-transmitters in turn can stimulate other cells (neurons or astrocytes) in the spatial vicinity^10^,^11^.

While the specific dynamics of network elements can take different forms, we show that, depending on the connectivity of the network, the active network transport and the passive environmental transport sometimes cooperate in spreading the excitation throughout the network and at other times compete, effectively inhibiting propagation through the system.

**Figure 2:**
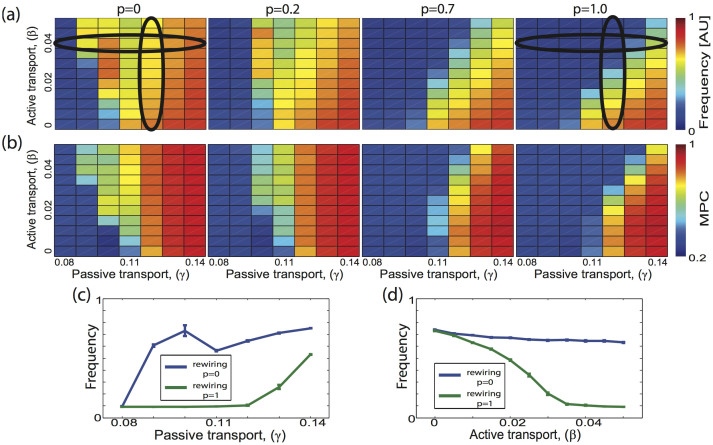
Changes in mean nodal frequency (a) and mean phase coherence (b) as a function of relative strength (amplitude) of both transport processes (x-axis - strength of interaction of the nodes with the physical space *γ*, y-axis - efficacy of network connectivity *β*) and network topology. Every location represents results averaged over 9 simulation runs. (c) Changes of nodal mean frequency for local (blue) and random (green) network topologies as a function of passive transport amplitude (*γ*); *β* = 0.04. (d) Changes of mean frequency as a function of the active transport amplitude (*β*) for local (blue) and random (green) network topologies; *γ* = 0.12.

We investigate the spatio-temporal pattern formation within the network as a function of the network topology, the strength of network connections, and the feedback amplitude between the environment and the network nodes. It is well established that if the magnitude of either network coupling or environmental feedback amplitude is large enough then that process alone can drive the formation of sustained large-scale spatio-temporal patterns^12^,^13^. However, we observe that for intermediate coupling values of both processes a strong dependence on network topology emerges. For local network topologies the two transport mechanisms cooperate, forming globally propagating waves, while for random topologies the processes compete, impeding signal propagation throughout the system. This effect is shown on Fig. 2, which shows the mean nodal frequency and mean pairwise phase coherence (MPC) between network nodes as a function of the magnitude of the feedback between the environment and the nodes (*γ*), and efficacy of network connectivity (*β*) for four different network topologies. When the network is effectively disconnected (*β* → 0) and *γ* ≤ 0.11 the mean frequency of nodal activity is low as it is driven only by external noise. When a certain amplitude (*γ* = 0.11) of nodal interaction with the environmental agent is reached the network enters a high activity global activation state (Fig. 2). However, if the amplitude of network coupling is increased for local network topologies (*p* = 0) the transition to sustained global activation of the network takes place for lower values of *γ* (Figs. 2a, 2c) - the two transport processes cooperate. In contrast, for global network topologies (*p* = 1) sustained network activation is significantly delayed as *γ* increases (Figs. 2a, 2c) - the two transport processes compete, impeding each other. Figures 2c and 2d summarize the difference in network activation for the two topologies as a function of both the magnitude of network transport and the environmental feedback. The increase of network coupling can play reverse roles in affecting the global network activation depending on the network topology.

**Figure 3:**
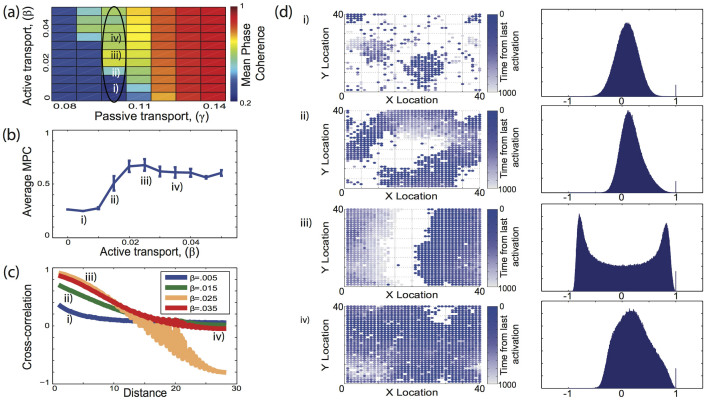
Formation of global activity patterns in networks with local connectivity. (a) Changes of the mean phase coherence as a function of active and passive transport amplitudes. (b) Mean phase coherence as function of active transport amplitude for *γ* = 0.10. (c) Mean cross-correlation between pairs of nodes as a function of distance within the network. (d) Examples of network activation at a time t (the difference between the current time and the time of the last nodal activation), and histograms of pairwise cross-correlation values for different active transport amplitudes (as denoted in a).

For local network topologies the cooperation of the two processes is due to the fact that both processes support local signal propagation from a recently activated node to its neighbors. Active network transport is faster (it is instantaneous) than environmental diffusion. However, on short spatial scales, the excitation stemming from the two processes can partially coincide providing supra-threshold excitation and leading to activation of new nodes in the proximity of the active site. As this process continues away from the originating site it forms large scale propagating waves within the network. The formation of local patterns that propagate through the network depends on the interplay between the magnitude of the diffusion constant, *D*, and the signal decay constant *α* (see Eqn. (1) in Methods section). The transition to global activation patterns as a function of the magnitude of network coupling is depicted in Fig. 3d, where we show co-activation patterns between network nodes giving global and local changes of mean phase coherence and cross-correlation in network activation sites. For weak network coupling (Fig. 3, location i), diffusion itself can not sustain large enough areas of local activation for the signal to spread globally through the network. Instead, random local domains are formed and then dissipate quickly. This leads to relatively low correlation between network nodes (Fig. 3b). The mean size of the formed domains can be inferred from the distance dependence of cross correlations between the nodal activation patterns (Fig. 3c). Characteristic network activity plots show the evolving size of synchronous excitations (Fig. 3d, left row). Histograms of pairwise cross-correlation values are Gaussian due to the stochasticity of the activated locations (Fig. 3d, row i right).

For intermediate levels of network coupling (locations ii and iii in Fig. 3), the domains spread through the network forming first irregular large scale activation patterns (Fig. 3, location ii), and then organizing into propagating waves (Fig. 3; location iii). Large irregular areas of activation lead to slower spatial decay of the cross-correlations (Fig. 3b), and a significant skew toward positive cross-correlation values on the pairwise histogram (Fig. 3d, row ii right). Formation of regular propagating waves through the network (Fig. 3, location iii), is characterized by strong correlation of nodal activity in the direction normal to the wave propagation, and anti-correlation in the direction of wave propagation (Fig. 3b, d; location iii). These regular waves result from the front of activation closely following nodes which have recently recovered from the previous cycle of activity. The match between the nodal refractory time and the speed of the wave propagation also explains the observed non-monotonic behavior of the mean nodal frequency (Fig. 2a). Further strengthening of the coupling of either process increases the speed of network activation, which in turn leads the excitation to self-extinguish due to the nodal refractory time. The activation needs then to be randomly restarted which leads to a reduction of the mean nodal frequency. The non-monotonicity resulting from the size of the environment does not change the overall observation that global network activation occurs at lower coupling strengths for local network connectivities as opposed to higher coupling values for random ones.

Finally, when the network coupling is high the whole network activates quickly (Fig. 3d; location iv). The location initiating the burst and the pattern of spread are random. Furthermore, anti-correlated islands may form if a given location was recently activated because of the noise and is in its refractory state.

**Figure 4:**
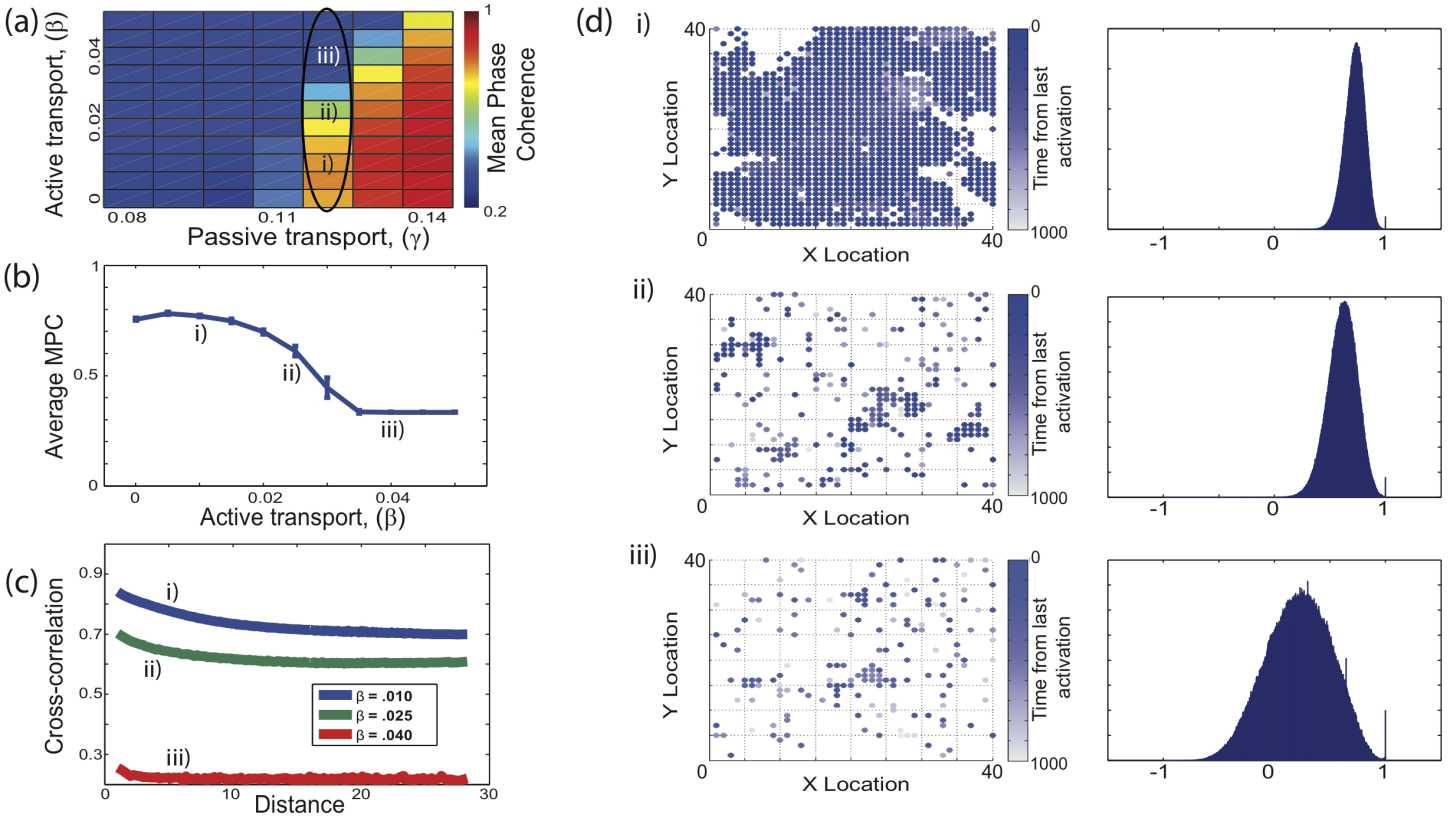
Formation of global activity patterns in networks with random connectivity. The individual panels correspond to those in Fig. 3.

For random network topologies (rewiring probability *p* = 1) increases in network connectivity strength have the opposite effect. Even for relatively strong interactions with the environment, the network is unable to support global activation patterns. The high level of connectivity drives the network towards homogeneity. The agent accumulated locally will quickly be transported to other locations, effectively reducing the local activation and preventing the nodes from activating. Only when the external feedback is strong enough to counteract this dissipation can the network support sustained activation (Fig. 4), leading to competition of the two processes. The network effectively supports only two modes of activity: random with formation of small domains (Fig. 4a locations ii and iii) and fully synchronous (Fig. 4a location i).

**Figure 5:**
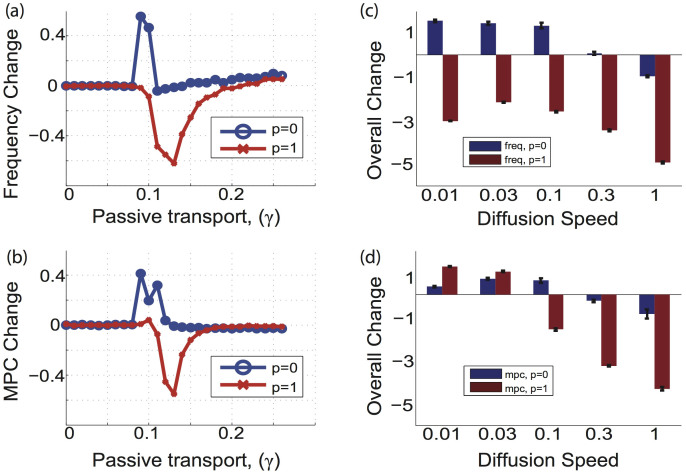
Cooperation and competition of active and passive processes as a function of changes in frequency and mean phase coherence (MPC) as functions of diffusion speed, D*. (a) Change in frequency between *β* = 0.05 and the baseline (*β* = 0) as a function of passive transport strength. (b) Change in MPC between *β* = 0.05 and the baseline (*β* = 0) as a function of the passive transport strength. In panels a and b positive change denotes cooperation, while negative values denote competition. (c) Total increase and/or decrease in the frequency across all values of *γ* due to the active process (calculated as a area under the curves in (a)). (d) Total increase and/or decrease in the MPC across all values of *γ* due to the active process (calculated as a area under the curves in (b)).

We also investigated how the diffusion constant, D*, affects the degree of cooperation or competition in the system. To do this we subtracted the mean frequency, or mean phase coherence, for the active process case with no coupling (*β* = 0.00) from that with strong coupling (*β* = 0.05), Fig. 5a, b. Comparing this baseline change for both metrics allows us to elucidate the extent to which the active process advances or delays changes in the frequency and MPC due to an increase in diffusion speed. We calculated the total area under the curves in Fig. 5a and b for all investigated values of *γ*, Fig. 5c, d.

The competition and cooperation patterns vary for both quantities and for both local and random network topologies. For local network connectivity, lower diffusion speeds lead to overall cooperation, whereas higher ones lead to competition. The general pattern of change in this case is similar for both frequency and MPC. The situation is different for networks with random connectivity. Here, the frequency shows competition for all of the speeds tested but its magnitude is non-monotonic with the diffusion speed (Fig. 5c). At the same time, for random topologies, the degree of cooperation and competition as reported by mean phase coherence changes largely monotonically as a function of the diffusion speed (Fig. 5d).

The complex interaction of the two processes can be attributed to matched, for cooperation, or mismatched, for competition, patterns of agent spread, as supported by the two processes. The local spread of diffusion is defined by its speed, which determines the spatial range and the time scale within which the local agent level is affected. Network connectivity, on the other hand, determines the ratio of the local to global signal spread through the system. While the transmission speed here is largely instantaneous, the effective speed of signal spread depends on actual activation levels of the nodes; that is, i.e. single activation of a node by the active process usually is not enough to trigger activation of a given site and is contingent on further agent increase from other sources. Thus if the spread of passive and active processes match in their spatial scope and the timescale, then the two processes cooperatively may lead to activation of subsequent network sites far from the original one. This however, due to the intrinsic properties of diffusive signal spread, may only happen for local network topologies.

Competition arises when the activation of the nodes by one process is impeded by their earlier activation by the other process. This is predominantly (but not only) observed for random topologies. In this case the spatially random activation of nodes can be driven by two processes - noise and active spread from other sites through random connectivity. While both processes are independent of each other, activation of a given site (due to noise or active spread) and subsequent local activation of its neighbors due to passive agent spread through diffusion precludes instantaneous activation of that site due to the nodal refractory time since a node will not fire within a predefined time window after its activation. Thus the two processes compete for access to available nodes limiting the overall activation frequency.

## Discussion

We have shown that network topology together with feedback from the external environment in which the network is embedded, may constitute a determining factor for the creation of sustained large-scale spatio-temporal patterns in a excitable system. We showed that local network topologies may cooperate with passive environmental transport to create globally propagating excitation through the system, whereas for random network topologies the two processes compete, increasing significantly the threshold in magnitude of the environmental transport needed for global patterns to occur. These results should be of importance in any system where the interaction of the network with its embedding environment can not be neglected.

The work was supported by NSF CMMI grant no.1029388.

## Methods

### Model

The spread of the agent is governed by two mechanisms: active spread through network connectivity, and passive environmental diffusion. The model describing the network nodes is very simple and is limited to charging and discharging elements that communicate through rapid equalization of the concentrations of the active agent. Such elements are relatively common in biology, and the model presented here roughly corresponds (to but is not limited) to astrocytic interactions. Passive diffusion is a natural choice for interactions through the environment as this is a common mode of environmental transport. Selecting diffusion as the interaction mechanism in the network requires the addition of a release like mechanism, which is accomplished by having an agent flush which is assumed to originate from an internal storage (Fig. 1).

The equation describing the nodal dynamics is a modified integrate-and-fire model^14^: 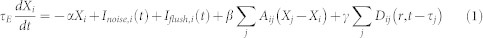
*X_i_* is the amount of active agent within the *i*-th node. The element time constant is set to *τ_E_* = 3, while the nodal leak constant is set to *α* = 1. The *I_noise_*_,*i*_ is an instantaneous sub-threshold release defined as 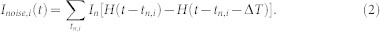
Here, *H* is the Heaviside function, the noise amplitude *I_n_* = 2.1; *t_n_*_,*i*_ denotes a specific noise instance; Δ*T* = 1 is the pulse duration. The noise arrives randomly at a given node with probability *p_n_* = 0.01. *I_flush_*_,*i*_ describes the internal auto-release current when the level of the agent reaches the threshold, *X_i_* = 1: 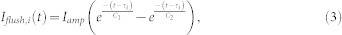
where *τ_i_* denotes the timing of the last threshold crossing in simulation steps. The size of the internal release is set to *I_amp_* = 1.5. The wave form is modeled as the difference between a slow, *C*_1_ = 300, and a fast, *C*_2_ = 30, exponential. Note that *I_flush_* has a strong positive phase shortly after its activation which decays away over the duration of its refractory period. The refractory period, 1000 timesteps, prevents continuous activation of the system.

The final term on the RHS describes the effect of the external, or environmental, excitation arriving at each network element. *γ* is the coupling amplitude, and *D_ij_*(*r*, *t* − *τ_j_*) is the solution of the two dimensional diffusion equation at a distance *r* and time *t* in the space embedding the network. 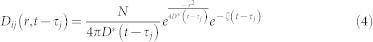
For most of the simulations (except figure 5) *N* = 100 is the amplitude of neurotransmitter release. The diffusion constant is D* = 0.1, and the time decay constant of the agent in the external environment is *ζ* = 0.01. For simplicity we assume that the element releases the agent into the physical space only when its value is supra-threshold.

We use a Small World paradigm^15^ to vary the network connectivity; initially the connections are set locally within given radius and then randomly rewired with probability *p*. The nodes are coupled through diffusive coupling with efficacy *β*, with the connection from element *j* to element *i* denoted by *A_ij_* taking the value 0 or 1. This paradigm allows for the range of network connectivity structures, from local to random connections, to be controlled by the rewiring parameter, *p*. The strength of the active transport (*β* = 0.00–0.08) is limited by the amount of excitatory agent that it is capable of sending out at any moment, as well as how much a given element retains information of its past. The overall dynamics of the system strongly depends on the amount of the diffusive agent and its feedback interaction with the nodes. The parameters are chosen so that small network and environmental interactions result in a quiescent network while large environmental feedback results in hyperactivity of the nodes. The parameters near this transition were of interest (*γ* = 0.08–0.14).

### Simulations

The model was simulated on a two dimensional network consisting of 1600 oscillators on a 40 × 40 grid with periodic boundary conditions. The active connections on the network are initially assigned a radius *R* = 2. The network was initialized with no activity or charge present and allowed to evolve for 50,000 timesteps using Euler's method. The initial 10,000 timesteps were removed from the analysis to allow the network to transition away from its (random) initial conditions. The figures displayed are analyzed over nine runs of the simulations for each set of parameters (*β*, *γ*, *p*).

### Analysis

The mean nodal frequency is calculated as the number of times any oscillator surpasses the charging threshold divided by the product of the number of oscillators and the timesteps in the simulation and then normalized. A frequency of one corresponds to a maximal raw firing frequency which is limited by the refractory time, 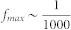. The units are not tied to actual timescales due to the generality of the model.

The mean phase coherence^16^ measures the degree of locking between nodal activations, and is calculated from the relative nodal activation times. The pairwise mean phase coherence is defined by: 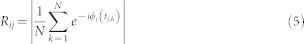
Where *ϕ_i_*(*t_j_*_,*k*_) is the instantaneous phase of the *j*-th node relative to the *i*-th node at *j*'s activation at time, *t_j_*_,*k*_, 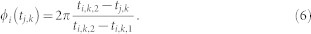
Here *t_j_*_,*k*_ is the *k*-th activation time of node *j* and *t_i_*_,*k*,1_, *t_i_*_,*k*,2_ are bracketing activation times of *i*-th node such that *t_i_*_,*k*,1_ ≤ *t_j_*_,*k*_ ≤ *t_i_*_,*k*,2_. The network wide mean phase coherence is the arithmetic average of the pairwise mean phase coherence.

## Author Contributions

D.M. and M.Z. designed the model and wrote the manuscript. D.M. performed the computer simulation, M.Z. provided theoretical guidance. Both authors reviewed the manuscript.

## References

[b1] Newman M. E. J. The structure and function of complex networks. SIAM Rev. 45, 167–256 (2003).

[b2] Newman M. E. J. Communities, modules and large-scale structure in networks. Nat. Phys. 2531 (2011).

[b3] Albert R. & Barabási A.-L. Statistical mechanics of complex networks. Rev. Mod. Phys. 74, 47–97 (2002).

[b4] Barthelemy M. Spatial networks. Phys. Rep. 499, 1–101 (2011).

[b5] Bullmore E. & Sporns O. Spatial networkscomplex brain networks: graph theoretical analysis of structural and functional systems. Nat. Rev. Neurosci. 10, 186–198 (2009). 19190637 10.1038/nrn2575

[b6] Zipf G. Human behaviour and the principle of least effort (Addison-Wesley, Cambridge, MA, 1949).

[b7] Li G. *et al.* Towards design principles for optimal transport networks. Phys. Rev. Lett. 104, 018701 (2010). 20366398 10.1103/PhysRevLett.104.018701

[b8] Ghoshal G., Sander L. & Sokolov I. {SIS} epidemics with household structure: the self-consistent field method. Math. Biosci. 190, 71–85 (2004). 15172803 10.1016/j.mbs.2004.02.006

[b9] Dupont G. & Goldbeter A. One-pool model for ca2+ oscillations involving ca2+ and inositol 1,4,5-trisphosphate as co-agonists for ca2+ release. Cell Calcium 14, 311–322 (1993). 8370067 10.1016/0143-4160(93)90052-8

[b10] Gibson W., Farnell L. & Bennett M. A quantitative model of atp-mediated calcium wave propagation in astrocyte networks. In Deutsch, A. *et al.* (eds.) Mathematical Modeling of Biological Systems, Volume II, Modeling and Simulation in Science, Engineering and Technology, 193–204 (Birkhuser Boston, 2008).

[b11] Postnov D., Koreshkov R., Brazhe N., Brazhe A. & Sosnovtseva O. Dynamical patterns of calcium signaling in a functional model of neuron-astrocyte networks. J. Biol. Phys. 35, 425–445 (2009). 19669421 10.1007/s10867-009-9156-x PMC2750744

[b12] Torre V. Synchronization of non-linear biochemical oscillators coupled by diffusion. Biol. Cybern. 17, 137–144 (1975). 1125340 10.1007/BF00364162

[b13] Bin D., Jiang W. & Xiangyang F. Chaotic synchronization with gap junction of multi-neurons in external electrical stimulation. Chaos Soliton. Fract. 25, 1185–1192 (2005). 10.1109/IEMBS.2005.1616874 17282643

[b14] Burkitt A. A review of the integrate-and-fire neuron model: I. homogeneous synaptic input. Biol. Cybern. 95, 1–19 (2006). 16622699 10.1007/s00422-006-0068-6

[b15] Watts D. & Strogatz D. Collective dynamics of small-world networks. Nature 393, 440 (1998). 9623998 10.1038/30918

[b16] Mormann F., Lehnertz K., David P. & Elger C. E. Mean phase coherence as a measure for phase synchronization and its application to the {EEG} of epilepsy patients. Physica D 144, 358–369 (2000).

